# Effect of flaxseed oil supplementation on anthropometric and metabolic indices in patients with coronary artery disease: A double-blinded randomized controlled trial

**DOI:** 10.15171/jcvtr.2019.26

**Published:** 2019-06-30

**Authors:** Sevda Saleh-Ghadimi, Sorayya Kheirouri, Ali Golmohammadi, Jalal Moludi, Hamed Jafari-Vayghan, Mohammad Alizadeh

**Affiliations:** ^1^Faculty of Nutrition and Food Sciences, Tabriz University of Medical Sciences, Tabriz, Iran; ^2^Student Research Committee, Tabriz University of Medical Sciences, Tabriz, Iran; ^3^Cardiovascular Research Center, Tabriz University of Medical Sciences, Tabriz, Iran; ^4^Nutrition Research Center, Tabriz University of Medical Sciences, Tabriz, Iran

**Keywords:** Anthropometric indices, CAD, Flaxseed oil, Lipid profile

## Abstract

***Introduction:*** It has been established that omega 3 fatty acids have cardio-protective effects through modulation of cardiometabolic risk factors via multiple mechanisms. The aim of this study was to investigate the effects of flaxseed oil on anthropometric indices and lipid profile in patients with coronary artery disease (CAD).

***Methods:*** A randomized, double-blind, placebo-controlled trial was performed in 44 patients with CAD. The subjects were randomly assigned to receive either 200 ml of 1.5% fat milk supplemented by 5 g of flaxseed oil (containing 2.5 g α-Linolenic acid) as intervention or 200 ml of 1.5% fat milk as placebo group for 10 consecutive weeks. Anthropometric indices and lipid profile were assessed at baseline and post-intervention.

***Results:*** The results indicated that supplementation with flaxseed oil had no impact on anthropometric indices. Weight, body mass index, waist circumference and hip circumference decreased statistically significant within groups, but not between groups. At the end of the intervention, diastolic blood pressure (DBP) decreased significantly (*P *= 0.022) in the intervention group. Moreover, the triglyceride (TG) level decreased significantly in the intervention group from 173.45 (49.09) to 139.33 (34.26) (*P *< 0.001). Other lipid profile indices including total cholesterol, low density lipoprotein and high density lipoprotein did not differ significantly within and between groups.

*** Conclusion:*** We observed that supplementation of flaxseed oil improved TG and DBP but had no effect on other lipid profiles and anthropometric indices in patients with CAD.

## Introduction

Cardiovascular diseases are the major cause of mortality worldwide, also in Iranian adults at present and will be the main health problem in the country in the future^1^ despite intensive management of risk factors and efforts to improve therapeutic approaches.^2,3^ In addition, the prevalence of many conventional risk factors of coronary artery disease (CAD) such as low physical activity and abdominal obesity are high in developing countries.^4,5^ The association between anthropometric indices and lipid profile with CAD has been well documented.^6,7^ Recent guidelines emphasize the necessity of controlling blood pressure, dyslipidemia, and reducing visceral fat.^8,9^ As a result, a complementary treatment is needed to lower several cardiometabolic risk factors. Data on herbal medicine have revealed promising properties of flaxseed oil in prevention and management of CAD.^10,11^

Flaxseed oil is belonging to one of the richest plant sources of the omega-3 fatty acids i.e. α-Linolenic acid (ALA), and is traditionally used for the treatment of several kinds of illnesses including inflammatory and neurodegenerative disorders.^12^ Recent studies have reported that administration of a Mediterranean ALA rich diet vs a usual diet is effective in reducing mortality after myocardial infarction (MI).^13,14^ Moreover, the effectiveness of flaxseed supplementation as an ALA source in modulation of cardiovascular risk factors has been investigated.^15,16^ It is assumed that the conversion of ALA to long chain polyunsaturated fatty acid (PUFA), eicosapentaenoic acid (EPA) and to some extent to docosahexaenoic acid (DHA) are involved in delivering these therapeutic effects.^17^

Flaxseed or its oil has been used in various products namely milk and dairy products for human consumption.^18^ Enrichment of foods with omega-3 provides more food choices for subjects who are trying to increase the omega-3 content in their diet. In a recent study adding 7% flaxseed oil to the skimmed milk did not have negative effects on physicochemical parameters of the emulsion. Moreover, the overall acceptability of the flaxseed oil enriched milk was good.^19^ On the other hand, milk is consumed frequently. It is easily processing, packaging and converting to other dairy products.^18^ Our rationale to choose 2.5 g ALA/day was based on recommendation for adequate intake (1.6 g/day for men and 1.1 g/day for women),^20^ as well as previous reports preferred recommendations for modest dietary consumption of ALA (2–3 g/d) for the primary and secondary prevention of coronary heart disease.^21^ Therefore, in the current study the effect of 10 weeks intervention with milk as a delivery system, containing 2.5% flaxseed oil on anthropometric indices, lipid profile and blood pressure in CAD patients was determined.

## Methods and Materials

### Participants

Patients with CAD who were admitted to the Shahid Madani Medical Research and Training Center affiliated to Tabriz University of Medical Sciences (TBZMED), Tabriz, Iran, were enrolled to participate in the current study. They were eligible if they: 1. were willing to participate in the study; 2. were angiographically confirmed as CAD which is defined as presence of at least 50% stenosis in at least one of the major coronary vessels; 3. aged 30 to 65 years old; and 4. had body mass index (BMI) between 25 to 35 kg/m^2^. Exclusion criteria included development of an MI in previous six months, clinical diagnosis of heart failure (function class III and IV), heart valve disease, uncontrolled diabetes, cancer, chronic inflammatory disease and autoimmune disease, using immunosuppressive drugs, weight loss drugs and food supplements; fish oil or (omega-3) fatty acid supplements use at the time of the study, drug users; pregnant and lactating women and lactose deficient subjects (milk intolerance).

### Study design

This was a double blind randomized controlled trial. The consent form that was approved by TBZMED completed by eligible participants. The method of participants’ recruitment was through announcement flyers in 2 centers: Sheikhorraees clinic and Tabriz Shahid Madani hospital. Eligible participants entered the study via simple sampling and they were randomly assigned in equal proportion either the intervention or the placebo group. The sequence of random allocation in each block was generated using a random sequence generator software, consisted 4 subjects per block. The criteria for matching were sex, age, BMI and medication received type/dose. The random sequence was kept and administered by a third investigator until all the outcome data collection was completed.

### Intervention

Fresh unrefined organically grown cold press flaxseed oil was purchased from a local supplier. We decided to use a delivery system for flaxseed oil supplementation to increase the compliance and acceptability. Based on previous studies, milk is considered as a suitable substance to prepare a stable emulsion.^19,22^ The process of emulsion preparation and packing were done in Pegah Dairy Co., Tabriz, Iran via high pressure homogenization method. Every intervention packet contained 200 mL sterilized 1.5% fat milk fortified with 2.5% flaxseed oil and the control packets contained 200 mL sterilized 1.5% fat milk. The packets were encoded as “A” and “B” in the industry to create the blinding and conceal the random allocation. Unbinding was only occurred once all the outcome data collection was completed. The vanilla essence was added to both intervention and placebo samples to maintain blinding process. The compliance was checked by phone calls every week. The milk packets were given to the subjects every two weeks. The participants were asked to record the number of unused milk packets in a case report form. For ethical issues, the subjects were permitted to consume their routine medications. However, taking any antioxidants and/or vitamin supplements; fish oil or other (omega-3) fatty acid supplements was prohibited during the trial. To adjust the effect of diet on the study outcomes, all the participants received a moderate calorie restricted dietary plan during ten weeks intervention period. A trained dietitian estimated the energy requirements and macronutrient distribution and trained participants on the diet. The participants were allowed to discontinue the trial if they were unwilling to complete or experience any adverse effect during the intervention. Safety assessment was done during the intervention by interviewing the participants and evaluating for any adverse effects related to the trial.

### Measurements of anthropometric indices, blood pressure, food intake and physical activity

Anthropometric indices including height, weight, waist circumference (WC), and hip circumference (HC) were measured at baseline and end of the intervention. Digital column scale coupled with a stadiometer were used to measure weight and height. WC and HC were determined via a non-elastic tape. Blood pressure was measured after 10 minutes rest, twice by 5 minutes interval between measurements in a sitting position. Food intake was assessed using a three days food record method at baseline and ten weeks later. Nutritionist IV was used to analyze the total energy and macronutrients intake. The physical activity level of participants was determined using international physical activity questionnaire (IPAQ). The IPAQ instrument assesses multiple domains of activity in addition to leisure time physical activity during last seven day.^23^

### Blood collection and measurement of biochemical indices

At baseline and after 10 weeks, blood samples were taken after 10-12 hours fasting and centrifuged immediately (3500 g, 10 minutes). The serum was stored at -80°C until further analysis. An enzymatic assay was used to measure fasting blood sugar (FBS), total cholesterol (TC), high density lipoprotein (HDL), and triglyceride (TG) (Pars Azmoon, Tehran, Iran). The Friedewald formula was used to estimate low density lipoprotein (LDL) levels.

### Statistical analysis

SPSS statistics software, version 21 was used for statistical analysis. Distribution of data was examined with the Kolmogorov–Smirnov expressed as mean (SD) for normally distributed quantitative data and frequency (percent) for qualitative data. The chi-squared test and the independent samples t-test was used to compare the two groups for baseline measures of the qualitative and quantitative data, respectively. Comparison of the two groups at the end of the study was completed by analysis of covariance (ANCOVA) after adjusting for the baseline parameters and covariates. Comparisons between baseline data and final results within each group was made by paired samples t-tests. Results with *P* values of <0.05 was considered statistically significant.

## Results

### Baseline characteristics of participants

**Figure 1:**
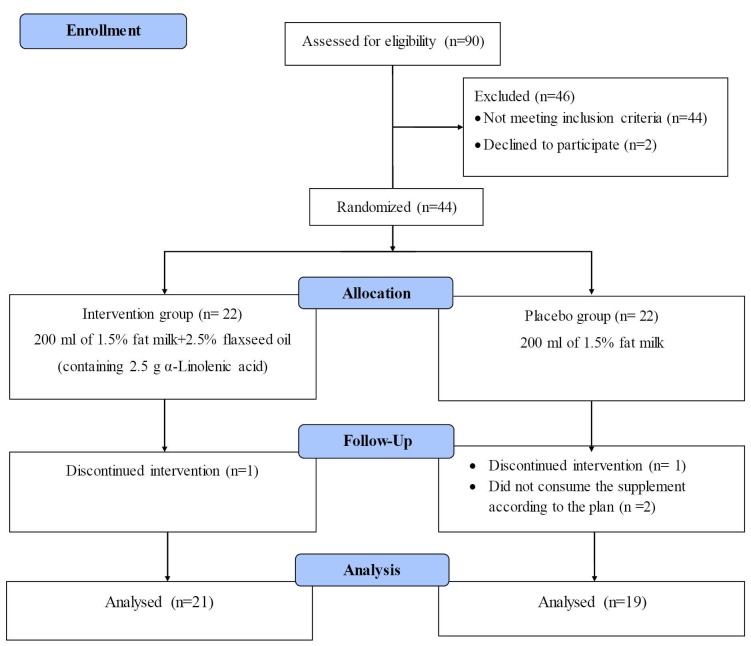
Flowchart of the study.

The CONSORT flowchart of the study is represented in Figure 1. Out of a total of 44 patients, 40 completed the trial (intervention group, n = 21; placebo group, n= 19). The baseline characteristics of the patients are shown in Table 1. The mean (SD) age of participants were 55.67 (6.89) and 54.79 (7.80) years in intervention and placebo groups, respectively with no statistically significant difference among them (*P* > 0.05). Of the patients, 90.5% and 89.5% were male in intervention and placebo groups, respectively. No significant difference was seen in other baseline parameters including sex, duration of CAD, family history of CAD, smoking and physical activity level between 2 groups (*P* > 0.05).

**Table 1:** Baseline characteristics of the study subjects^a^

**Variable**	**Flaxseed oil (n=21)**	**Placebo** **(n=19)**	***P*** **value** ^c^
Age (y)^b^	55.67 (6.9)	54.80 (7.8)	0.708^d^
Sex			
Male	19 (90.5)	17 (89.5)	1.00
CAD duration (y)^b^	2.6 (1.1)	2.8 (1.7)	0.618^d^
Family history of CAD	13 (61.9)	12 (63.2)	0.935
Smoking	1 (4.8)	3 (15.8)	0.331
Physical activity			
Low	8 (38.1)	5 (26.3)	0.591
Moderate	12 (57.1)	14 (73.7)	
High	1 (4.8)	0 (0.0)	

^a^Values are expressed as No. (%). ^b^Values are expressed as mean (SD). ^c^Chi-square test. ^d^Independent samples *t* test.

### Dietary intake

Dietary intakes of energy and macronutrients are demonstrated in Table 2. There were no statistically significant differences in energy and macronutrients intake between the 2 groups at baseline and after 10 weeks intervention (*P* > 0.05). Energy and macronutrients intakes within group were significantly lower at the end of trial compared to the baseline (*P* < 0.05).

**Table 2:** Dietary intake of subjects before and after intervention

**Variable**	**Flaxseed oil (n=21)**	**Placebo (n=19)**	**MD (95% CI)**
Energy (Kcal/d)			
Before	2125.88 (280.19)	2099.95 (296.42)	-25.93 (-210.54 to 158.66), 0.778^a^
After	1604.79 (198.16)	1620.58 (162.85)	29.02 (-41.19 to 99.24), 0.408^b^
MD (95% CI)^c^	-521.09 (-593.17 to -449.02), <0.001	479.37 (-574.21 to 384.52), <0.001	
Carbohydrate (g/d)			
Before	292.31 (38.53)	288.74 (40.76)	-3.56 (-28.95 to 21.82), 0.780^a^
After	216.06 (23.88)	222.83 (22.39)	8.45 (-17.43 to 0.53), 0.064^b^
MD (95% CI)^c^	-76.25 (-86.80 to -65.70), <0.001	-65.91 (-78.95 to -52.87), <0.001	
Protein (g/d)			
Before	79.73 (10.51)	78.75 (11.17)	-0.98 (-7.90 to 5.95), 0.777^a^
After	58.39 (7.30)	60.77 (6.10)	2.38 (-0.15 to 4.91), 0.070^b^
MD (95% CI)^c^	-20.86 (-23.71 to -18.00), <0.001	-17.98 (-21.53 to -14.42), <0.001	
Total fat (g/d)			
Before	70.86 (9.34)	70.00 (9.88)	-0.86 (-7.02 to 5.29), 0.700^a^
After	51.91 (6.49)	54.02 (5.43)	2.07 (-0.1 to 4.25), 0.061^b^
MD (95% CI)^c^	-18.51 (-21.05 to -15.96), <0.001	-15.98 (-19.14 to -12.82), <0.001	

MD: mean difference. Values are expressed as mean (SD) ^a^ Independent samples t-test; ^b^ Adjusted for baseline values using the analysis of covariance (ANCOVA) test;^c^Paired-samples *t* test.

### Blood pressure and anthropometric indices

As shown in Table 3, regarding weight, BMI, WC, HC and waist to hip ratio (WHR), no statistically significant difference was observed between 2 groups at baseline and after intervention (*P* >0.05). After 10 weeks flaxseed oil supplementation, changes in weight, BMI, waist and hip circumference were found to be significant within groups. No significant change in systolic blood pressure (SBP) was seen within and between 2 study groups at baseline and after supplementation adjusting for confounding factors. However, diastolic blood pressure (DBP) had a significant decline in intervention group from 75.72 (8.51) to 72.70 (7.15) mm Hg; (*P* < 0.001). A significant change was observed between two study groups after adjusting for confounders (*P* =0.022).

**Table 3:** Effect of flaxseed oil supplementation on anthropometric indices and blood pressure

**Variable**	**Flaxseed oil (n=21)**	**Placebo (n=19)**	**MD (95% CI), P-value**
Weight (kg)			
Before	86.02 (10.70)	85.66 (10.19)	- 0.36 (- 7.18 to 6.45), 0.915^a^
After	83.85 (10.90)	83.66 (10.53)	-0.026 (-0.78 to 0.73), 0.944 ^b^
MD (95% CI), P-value^c^	-2.18 (-2.75 to -1.60), <0.001	-2.10 (-2.74 to -1.46), <0.001	
BMI (kg/m^2^)			
Before	30.36 (3.04)	30.70 (3.90)	0.34 (-1.95 to 2.62), 0.766 ^a^
After	29.58 (3.19)	29.93 (3.86)	0.024 (-0.26 to 0.31), 0.861 ^b^
MD (95% CI), *P* value^c^	-0.78 (-0.99 to -0.57), <0.001	- 0.77 (-0.98 to -0.55), <0.001	
Waist circumference (cm)			
Before	104.42 (7.68)	107.29 (8.63)	2.87 (-2.74 to 8.48), 0.305 ^a^
After	102.58 (7.92)	105.35 (8.99)	0.28 (-0.73 to 1.28), 0.581 ^b^
MD (95% CI), *P* value^c^	-1.84 (-2.63 to -1.05), <0.001	-1.94 (-2.56 to -1.33), <0.001	
Hip circumference (cm)			
Before	107.92 (6.80)	107.59 (16.06)	-0.33 (-8.72 to 8.06), 0.937 ^a^
After	106.61 (6.79)	106.44 (16.21)	-0.031 (-0.55 to 0.62), 0.914^b^
MD (95% CI), *P* value^c^	-1.31 (-1.73 to -0.89), <0.001	-1.15 (-1.76 to -0.54), 0.001	
WHR			
Before	0.97 (0.05)	1.03 (0.25)	0.06 (-0.06 to 0.18), 0.312 ^a^
After	0.96 (0.05)	1.02 (0.26)	0.018 (-0.18 to 0.15), 0.737 ^b^
MD (95% CI), *P* value^c^	**-**0.01 (-0.01 to 0.001), 0.119	-0.01 (-0.01 to 0.004), 0.253	
SBP (mm Hg)			
Before	116.62 (12.13)	112.50 (15.09)	-4.13 (-12.98 to 4.74), 0.352^a^
After	114.75 (13.02)	111.45 (17.33)	-1.96 (-11.01 to 7.09), 0.662^b^
MD (95% CI), *P* value^c^	**-**1.87 (-4.14 to 0.39), 0.814	**-**1.05 (-10.34 to 8.23), 0.100	
DBP (mm Hg)			
Before	75.72 (8.51)	79.59 (10.14)	3.86 (-2.36 to 10.08), 0.216^a^
After	72.70 (7.15)	78.88 (11.07)	2.42 (0.37 to 4.47), 0.022^b^
MD (95% CI), *P* value^c^	-3.02 (-4.52 to -1.52), <0.001	-0.71 (-1.89 to 0.48), 0.227	

MD: mean difference; BMI: Body mass index, WHR: Waist to hip ratio, SBP: Systolic blood pressure, DBP: diastolic blood pressure. Values are expressed as mean (SD) ^a^ Independent samples t-test; ^b^ Adjusted for baseline values using the analysis of covariance (ANCOVA) test;^c^Paired-samples *t* test.

### FBS and lipid profile

At baseline, no statistically significant difference was seen in FBS and lipid profile other than HDL between the groups (Table 4). At baseline, there was a statistically significant difference between groups in HDL level (*P*=0.038). Although, an increase was detected in HDL levels in both the groups as compared to baseline, supplementation with flaxseed oil did not alter HDL concentration significantly. At the end of the trial, a significant reduction was found in TG serum levels in the flaxseed oil group in comparison to baseline (*P*<0.001). Also, in the intervention group, TG serum levels declined statistically significant compared to placebo group (mean difference= 20.03, *P* =0.001).

**Table 4:** Effect of flaxseed oil supplementation on lipid profile and FBS status

**Variable**	**Flaxseed oil (n=21)**	**Placebo (n=19)**	**MD (95% CI),** ***P*** ** value**
FBS (mg/dL)			
Before	113.52 (44.76)	105.99 (23.87)	-7.53 (-30.86 to 15.79), 0.517^a^
After	107.35 (38.72)	111.42 (34.71)	11.45 (-2.37 to 25.27), 0.150^b^
MD (95% CI), *P* value^c^	-6.18 (-16.30 to 3.94), 0.218	5.43 (-3.84 to 14.69), 0.234	
TG (mg/dL)			
Before	173.45 (49.09)	164.09 (32.85)	-9.35 (-36.39 to 17.68), 0.488^a^
After	139.33 (34.26)	159.36 (37.68)	24.98 (9.54 to 40.43), 0.001^b^
MD (95% CI), *P* value^c^	-34.11 (-44.64 to -23.59), **<0.001**	-4.73 (-18.28 to 8.82), 0.473	
TC (mg/dL)			
Before	155.43 (32.04)	143.07 (22.63)	-12.35 (-30.29 to 5.58), 0.171^a^
After	150.62 (24.84)	143.74 (28.88)	-3.24 (-20.66 to 14.18), 0.820^b^
MD (95% CI), *P* value^c^	-4.81 (-18.34 to 8.72), 0.467	0.66 (-12.05 to 13.37), 0.914	
LDL (mg/dL)			
Before	88.77 (29.79)	83.21 (23.52)	-5.56 (-22.86 to 11.75), 0.520^a^
After	87.90 (28.20)	77.33 (25.98)	-8.12 (-33.79 to 17.54), 0.320^b^
MD (95% CI), *P* value^c^	-0.86 (-13.09 to 11.37), 0.885	-5.88 (-19.33 to 7.57), 0.370	
HDL (mg/dL)			
Before	31.98 (5.91)	27.66 (6.76)	-4.31 (-8.37 to -0.25), 0.038^a^
After	34.85 (8.67)	30.06 (6.49)	-2.23 (-7.26 to 2.80), 0.605^b^
MD (95% CI), *P* value^c^	2.88 (-0.95 to 6.70), 0.133	2.40 (-0.29 to 5.08), 0.077	

MD: mean difference. Values are expressed as mean (SD). ^a^ Independent samples t-test; ^b^ Adjusted for baseline values using the analysis of covariance (ANCOVA) test;^c^Paired-samples *t* test.

## Discussion

The present study evaluated the beneficial effects of flaxseed oil on anthropometric indices and its metabolic consequences in CAD individuals. Our results indicated that ten weeks supplementation with flaxseed oil had a significant effect on DBP and TG in patients with CAD. The main finding of this study would be that serum TG decreases as a result of consumption of milk with flaxseed oil.

The results showed that consumption of flaxseed oil would not be able to affect weight, WHR, WC and BMI significantly in the intervention group in comparison to placebo group. However, anthropometric indices decreased at the end of intervention in both intervention and placebo groups which could be because of calorie restricted diet. The findings are in agreement with results of Paschos et al, study who reported that supplementation with flaxseed oil in dyslipidaemic patients did not affect BMI.^11^ Also, in a study done by Kontogianni, et al,^24^ no differences were observed in weight after 6 weeks in young, healthy, normal weight adults after intervention by 15 ml/day of flaxseed oil. Although, flaxseed combination into the diet (40 g flaxseed/day) led to a significant decline in weight, BMI, WHR and WC in healthy menopausal women.^15^ Different dosages, duration of intervention and type of flaxseed oil, ALA sources, dietary intake, physical activity level and the clinical state of the participants may be some main reasons for observed diversity in the findings. The main mechanism of action for anti-obesity effects is not known. Based on current evidence, bioactive components of flaxseed oil, mainly ALA, may exhibit anti-obesity effects.^25^ Moreover, flaxseed oil contains numerous unsaturated fatty acids including linoleic acid and eicosadienoic in addition to ALA, all of which have anti-obesity effects.^26^ Also, the eicosanoids resulting from PUFA metabolism have ability to prevent adipocyte differentiation and induce apoptosis in preadipocytes.^27,28^ According to a meta-analysis, whole flaxseed rather than flaxseed-oil is effective in weight and BMI reduction^29^ which is attributed to the fact that the flaxseed can control the energy intake and increase satiety by containing 28% fibers. The non-significant results might be due to supplementation with oil seed rather than the whole one.

Supplementation with flaxseed oil led to a significant decrease (3 mm Hg) in DBP. A non-significant decrease was observed in placebo group which might be as a result of milk consumption. A recent meta-analysis revealed that milk proteins are slightly involved in lowering of blood pressure.^30^ Our study is in line with Sioen et al^31^ study who indicated that increased omega-3 intake especially ALA lowered the DBP significantly. Decrease in both SBP and DBP levels after supplementation with flaxseed oil has been reported in other studies.^11,32^ Recently, it was shown that ∼8 g/day ALA supplementation for 12 weeks decreased both DBP and SBP in dyslipidaemic men.^11^ On the contrary, in a trial conducted in hypertensive men and women, high dose of ALA (∼38 g/day) for 2 weeks did not significantly affect blood pressure.^33^ Several confounding factors including the type of subjects, visit-to-visit-variability of blood pressure, method for blood pressure measurements and dietary changes might have a role in consistency of the results. The main mechanisms by which flaxseed oil supplementation might lower blood pressure are not well known, but the action of prostaglandin metabolism on modulation of blood pressure may be involved. Prostaglandin may act by direct effects on vascular reactivity, regulation of renin release and control of sodium and water balance. As well, prostaglandin has vasodilator actions on control of renal blood flow and peripheral sympathetic tone and may influence the baroreceptors and cardiac output.^11,34,35^

In the current study we found a significant reduction in serum TG, indicating the cardioprotective property of flaxseed oil. Other lipid parameters were not changed. Previous studies reported different results considering flaxseed oil effect on lipid profile. Based on Dodin et al study, daily consumption of flaxseed (40 g) decreased LDL-c, TC and TG lipoprotein and increased HDL-c concentrations in healthy menopausal women after 12 months.^16^ The findings of the study by Kontogianni et al revealed that after 6 weeks of receiving flaxseed oil (15 mL/day ALA), no decrease was observed in lipid profile compared to the same period of consuming Olive oil.^24^ The modulating effects of flaxseed oil (i.e., ALA) on lipid metabolism might have been related to improving lipid homeostasis at the adipose tissue-liver axis, increased fatty acid β-oxidation through up-regulation of peroxisome proliferator-activated receptor-α and down-regulation of sterol regulatory element-binding protein-1.^36-38^ Concomitantly, flaxseed oil reduces lipogenesis, so TG levels reduction is resulted.^39^

HDL status was not considered as the eligibility for the participation in the current study. Nevertheless, the control group had a higher level of HDL in baseline which led to not significant difference between the two groups after intervention. Moreover, greater decline in lipid concentrations upon intervention was observed in patients with higher baseline lipid levels. Additionally, based on the evidence that HDL levels are inversely associated with the incidence of CVD,^40^ the 9% increase in the HDL concentration observed in the current study, may decrease the CVD risk.

In a study done by Torkan et al, a significant reduction of TC, LDL and TG was observed following ingestion of 30 g raw flaxseed in hyperlipidemic subjects.^41^ However, flaxseed derived lignan supplementation did not improve hyperlipidemia in type 2 diabetic patients.^42^ Flaxseed oil at the dose of 3 g/day showed a remarkable effects by reducing LDL and increasing HDL in older adults.^43^ Change in lipid profiles may depend on intake form of flaxseed, sex and age of the subjects and their lipid values.^42^ Overall, some animal studies have attributed the lowering effect of flaxseed oil on the TG and cholesterol serum level to its ALA content.^37,44^ The others revealed that hypocholesterolemia effect of flaxseed results from interaction of its compounds, i.e. fiber, lignin complex and high amounts of ALA.^45,46^ In our study, the lack of a significant effect of flaxseed oil on lipid profile, may be partly due to the normal levels of this outcomes at baseline which might be because of statin therapy done in almost the whole subjects.

There are limited studies evaluating the effects of flaxseed oil on FBS.^10,24,47^ Our results are in line with previous studies. In one study, consumption of milled flaxseed or flaxseed oil (13.2 g/day) for 12 weeks in adults with type 2 diabetes did not influence glycemic control, or FBS.^10^ Meropi et al indicated that ALA failed to lower FBS in healthy adults supplied with 15 mL/day of flaxseed oil.^24^ In the present study, reduction of FBS in the intervention group was more than the placebo group although was not statistically significant.

This study has some limitations that should be addressed. First, other known risk factors of coronary heart disease such as apolipoprotein A and B and inflammatory markers were not measured. Moreover, the potential effects of flaxseed oil on endothelial marker were not examined. Relatively short intervention period and small sample size are considered as other limitations of the study. Monitoring the patients’ diets through designing a calorie restricted dietary plan with focus on amount and type of fat is the strengths of the current study. As well, physical activities and dietary intakes were checked before and after intervention. The novelty of the current study is related to the use of milk/flaxseed oil emulsion as intervention that can also be an applicable substitute for the daily milk consumption by the patients who suffer from CAD.

In conclusion, the findings of the present study suggest that flaxseed oil consumption by CAD patients is effective in reducing TG and DBP, and is expected to significantly reduce the overall CVD risk factors. Our study shows that milk enrichment by flaxseed oil can be used as a food delivery for increasing omega-3 fatty acids.

## Ethical approval

The protocol was approved by Medical Ethics Committee of TBZMED (No. IR.TBZMED.REC.1395.804). The trial was registered in the Iranian Registry of Clinical Trials (identifier: IRCT2016071211288N10; https://www.irct.ir).

## Competing interests

All authors declare no competing financial interests exist.

## Funding

This study was supported by a grant from TBZMED as a thesis for PhD degree of the first author (Grant No. D/51).

## Acknowledgment

The authors wish to acknowledge all the patients for their cooperation in performing this project, support of Mr. Jodeyri also other staff of Pegah Dairy Co., Tabriz, Iran for their help in milk/oil emulsion preparation and packing. The authors also thank TBZMED for their financial support.
